# Improved exponential type ratio estimator in double sampling for stratification

**DOI:** 10.1038/s41598-023-49772-0

**Published:** 2023-12-18

**Authors:** Anurag Gupta, Rajesh Tailor, Nitu Barod

**Affiliations:** School of Studies in Statistics, Vikram University, Ujjain, M.P. 456010 India

**Keywords:** Applied mathematics, Statistics

## Abstract

The objective of this research is to create a chain-ratio-type exponential estimator in order to estimate the finite population mean in double sampling for stratification. An estimator for population mean has been constructed based on the concept of chain-ratio estimators. The constructed estimator is compared to the standard unbiased estimator, as well as the other relevant existing estimators and conditions are shown to yield better results in terms of efficiency. To support the theoretical results the study has been done on both natural as well as simulated populations.

## Introduction

Stratified random sampling is a commonly used approach in sampling, In recent years, significant advancements have been made in the realm of stratified sampling estimators, with a particular focus on integrating innovative techniques like L-moments. L-moments, an extension of conventional moments, offer a robust approach for characterizing the shape and scale of probability distributions. When applied to stratified sampling, L-moments provide a nuanced understanding of the underlying data distribution within strata, enabling more accurate and efficient estimation of population parameters. The utilization of L-moments in stratified sampling estimators represents a cutting-edge methodology that enhances the precision of estimations, especially in scenarios where the data may exhibit non-normality or complex distribution patterns. (for instance see Hosking^1^ and Shahzad et al.^2^.

Stratified random sampling is particularly used when there is prior knowledge about the sampling frame and strata weights. However, in many situations, obtaining up-to-date information on strata weights can be challenging due to the addition or deletion of units to the population. For instance, studying the socio-economic status of people in a particular region becomes difficult and expensive due to factors like immigration, emigration, and other demographic changes that affect the strata sizes consequently strata weights.

To address this issue, double sampling for stratification is often employed as an alternative to stratified random sampling. In double sampling for stratification, a large sample is initially selected, which is then divided into homogeneous strata to estimate the strata weights. From each stratum, a sample is selected using simple random sampling without replacement, and both study and auxiliary variables are observed.

Double sampling for stratification is a widely used sampling design in forest and resource inventory, particularly in forest ecosystems. For instance, Lam et al.^3^ applied double sampling for stratification in monitoring sparse tree populations in Chinese forests. This approach is cost-effective and robust.

The concept of double sampling traces its origins to Neyman^4^, when he first developed it to gather data on strata weights in stratified sampling. Rao^5^ extended its application to address non-response issues and analytical comparisons. Ige and Tripathi^6^ proposed alternative sampling strategies based on double Sampling for Stratification (DSS), utilizing auxiliary information from the first-phase sample in both survey design and estimation.

This work led the way for Singh and Vishwakarma^7^ to introduce a general procedure for estimating population means using double sampling for stratification and auxiliary information. Tailor et al.^8^ built upon the foundation laid by Ige and Tripathi^6^ by exploring ratio-type and product-type exponential estimators.

For further research in this field, readers are encouraged to explore the papers by Tailor and Lone^9^, Singh and Nigam^10^, Gupta and Tailor^11^, Lone et al.^12^, and Verma et al.^13^.

Previous research has focused on classical ratio and product estimators for population mean in double sampling for stratification. Motivated by the aforementioned studies, this research introduces a novel approach by developing a chain ratio-type exponential estimator for estimating the population mean in double sampling for stratification. By exploring this new estimator, we aim to contribute to the existing literature and provide an alternative method for population mean estimation in double sampling for stratification.

## Procedure for double sampling for stratification and notations

Suppose $U = \left( {U_{1} ,U_{2} , \ldots ,U_{N} } \right)$ is a finite population of size N units, which consists of strata weights $\frac{{N_{h} }}{N},\,\left( {h = 1,2,3, \ldots ,L} \right)$. The weights of the population *U* are unknown. In this scenario double sampling for stratification will be used.

Procedure for double sampling for stratification:

- In the initial phase, a sample *S* of size *n'* is drawn using simple random sampling without replacement, and auxiliary variables *x* and *z* are recorded.
- The sample is then divided into *L* strata based on the observed variables *x* and *z*. Let $n_{h}^{\prime }$ denote the number of units in each stratum $\left( {h = 1,2,3, \ldots ,L} \right)$, such that $n^{\prime } = \sum\nolimits_{h = 1}^{L} {n_{h}^{\prime } }$.
- From each stratum with size $n_{h}^{\prime }$, a sample of size $n_{h} = v_{h} n_{h}^{\prime }$ is drawn, where $0 < v_{h} < 1,\left( {h = 1,2,3, \ldots ,L} \right)$. These predetermined probabilities $v_{h}$ determine the sample size $n_{h}$ from each stratum $n_{h}^{\prime }$. The combined sample S′ is obtained with a total size $n = \sum\limits_{h = 1}^{L} {n_{h} }$. In S′, both the study variable *y* and auxiliary variables *x* and *z* are observed. Let *y* be the study variable and *x* and *z* are first and second auxiliary variables respectively and $\overline{Y},\,\,\overline{X}\,$ and $\,\overline{Z}\,$ are population means of variables *y, x,* and *z* respectively

where $\overline{Y}\,\, = \frac{1}{N}\,\,\sum\nolimits_{h = 1}^{L} {\sum\nolimits_{i = 1}^{{N_{h} }} {y_{hi} } }$, $\overline{X}\,\, = \frac{1}{N}\,\,\sum\nolimits_{h = 1}^{L} {\sum\nolimits_{i = 1}^{{N_{h} }} {x_{hi} } }$ and $\overline{Z}\,\, = \frac{1}{N}\,\,\sum\nolimits_{h = 1}^{L} {\sum\nolimits_{i = 1}^{{N_{h} }} {z_{hi} } }$, $R_{1} = \frac{{\overline{Y}}}{{\overline{X}}}$, $R_{2} = \frac{{\overline{Y}}}{{\overline{Z}}}$, *R*_1_ and *R*_2_ are ratio of two population means, $\overline{Y}_{h} ,\,\,\overline{X}_{h} \,$ and $\overline{Z}_{h} \,$ are hth stratum mean for variable *y*, *x*, and *z* respectively

where $$\overline{Y}_{h} \,\, = \frac{1}{{N_{h} }}\,\sum\nolimits_{i = 1}^{{N_{h} }} {y_{hi} }$$, $$\overline{X}_{h} \,\, = \frac{1}{{N_{h} }}\,\,\sum\nolimits_{i = 1}^{{N_{h} }} {x_{hi} }$$ and $$\overline{Z}_{h} \,\, = \frac{1}{{N_{h} }}\,\sum\nolimits_{i = 1}^{{N_{h} }} {z_{hi} }$$,

$S_{yh}^{2} ,\,\,S_{xh}^{2} \,$ and $S_{zh}^{2} \,$ are hth stratum population variance of the variable *y*, *x*, and *z* respectively, where

$S_{yh}^{2} \,\, = \,\,\frac{1}{{N_{h} - 1}}\,\,\sum\nolimits_{i = 1}^{{N_{h} }} {\left( {y_{hi} - \overline{Y}_{h} } \right)^{2} }$, $S_{xh}^{2} \, = \,\frac{1}{{N_{h} - 1}}\,\,\sum\nolimits_{i = 1}^{{N_{h} }} {\,\left( {x_{hi} - \overline{X}_{h} } \right)^{2} }$ and $S_{zh}^{2} \, = \,\frac{1}{{N_{h} - 1}}\,\,\sum\nolimits_{i = 1}^{{N_{h} }} {\,\left( {z_{hi} - \overline{Z}_{h} } \right)^{2} }$, $S_{yxh} ,S_{yzh}$ and $S_{xzh}$ are hth stratum covariance between the variable $y$ and *x*, $y$ and *z*,* x* and *z* respectively, where

$$S_{yxh}^{{}} \,\, = \,\,\frac{1}{{N_{h} - 1}}\,\,\sum\nolimits_{i = 1}^{{N_{h} }} {\left( {y_{hi} - \overline{Y}_{h} } \right)\left( {x_{hi} - \overline{X}_{h} } \right)}$$, $$S_{yzh}^{{}} \,\, = \,\,\frac{1}{{N_{h} - 1}}\,\,\sum\nolimits_{i = 1}^{{N_{h} }} {\left( {y_{hi} - \overline{Y}_{h} } \right)\left( {z_{hi} - \overline{Z}_{h} } \right)}$$, and $$S_{xzh}^{{}} \,\, = \,\,\frac{1}{{N_{h} - 1}}\,\,\sum\nolimits_{i = 1}^{{N_{h} }} {\left( {x_{hi} - \overline{X}_{h} } \right)\left( {z_{hi} - \overline{Z}_{h} } \right)}$$,

$\overline{x}^{\prime } = \sum\nolimits_{h = 1}^{{n_{h} }} {w_{h} \overline{x}_{h}^{\prime } }$: First phase sample mean of auxiliary variable *x* which is unbiased estimator of $\overline{X}$,

$\overline{z}^{\prime } = \sum\nolimits_{h = 1}^{{n_{h} }} {w_{h} \overline{z}_{h}^{\prime } }$: First phase sample mean of auxiliary variable *z* which is unbiased estimator of $\overline{Z}$

$f = \frac{{n^{\prime } }}{N}$: Sampling fraction at first phase,

$$n = \sum\nolimits_{h = 1}^{L} {n_{h} }$$: sample size,

$w_{h} = \frac{{n_{h}^{\prime } }}{{n^{\prime } }}$: $hth$ First phase sample’s stratum weight,

$\nu_{h} = \frac{{n_{h} }}{{n^{\prime}_{h} }}$ Sampling fraction for sample units selected in second-phase sample.

$S_{y}^{2} \,\, = \,\,\frac{1}{N - 1}\,\sum\nolimits_{h = 1}^{L} {\sum\nolimits_{i = 1}^{{N_{h} }} {\left( {y_{hi} - \overline{Y}_{h} } \right)^{2} } } \,$: Population variance of the study variable $y$,

$S_{z}^{2} \,\, = \,\,\frac{1}{N - 1}\,\sum\nolimits_{h = 1}^{L} {\sum\nolimits_{i = 1}^{{N_{h} }} {\left( {z_{hi} - \overline{Z}_{h} } \right)^{2} } } \,$: Population variance of the auxiliary variable *z*,

$S_{yz}^{{}} \,\, = \,\,\frac{1}{N - 1}\,\sum\nolimits_{h = 1}^{L} {\sum\nolimits_{i = 1}^{{N_{h} }} {\left( {y_{hi} - \overline{Y}_{h} } \right)\left( {z_{hi} - \overline{Z}_{h} } \right)} } \,$: Population covariance between the variable $y$ and *z*,

## Some relevant existing estimators

The unbiased estimator for $\overline{Y}$ is defined as

3.1 $$\overline{y}_{ds} = \sum\nolimits_{h = 1}^{L} {w_{h} } \overline{y}_{h} ,$$

Cochran^14^ ratio estimator was studied in double sampling for stratification procedure by Ige and Tripathi^6^ and suggested a ratio estimator as

3.2 $$\hat{\overline{Y}}_{R}^{ds} = \overline{y}_{ds} \frac{{\overline{x}^{\prime } }}{{\overline{x}_{ds} }},$$

Using exponential function a ratio-type exponential estimator for $\overline{Y}$ was envisaged by Bahl and Tuteja^15^ in simple random sampling as

3.3 $$\hat{\overline{Y}}_{{\text{Re}}} = \overline{y}\,\exp \,\frac{{\left( {\overline{X} - \overline{x}} \right)}}{{\left( {\overline{X} + \overline{x}} \right)}},$$

Bahl and Tuteja^15^ estimator $\hat{\overline{Y}}_{{\text{Re}}}^{{}}$ was studied by Tailor et al.^8^ in double sampling for stratification procedure as

3.4 $$\hat{\overline{Y}}_{{\text{Re}}}^{ds} = \overline{y}_{ds} \exp \,\frac{{\left( {\overline{x}^{\prime } - \overline{x}_{ds} } \right)}}{{\left( {\overline{x}^{\prime } + \overline{x}_{ds} } \right)}},$$

Lakhre^16^ developed dual to ratio type exponential estimator in case of double sampling for stratification

3.5 $$\hat{\overline{Y}}_{{\text{Re}}}^{*} = \overline{y}_{ds} \exp \;\left( {\frac{{\overline{x}_{ds}^{*} - \overline{x}^{\prime } }}{{\overline{x}_{ds}^{*} + \overline{x}^{\prime } }}} \right),$$

where $$\overline{x}_{ds}^{*} = \frac{{N\;\overline{x}^{\prime } - n\;\overline{x}_{ds} }}{N - n}$$.

Lone et al.^17^ proposed the alternative of Ige and Tripathi^6^ estimator using the dual approach introduced by Srivenkataramana^18^ and Bandyopadhyay^19^ as

3.6 $$\hat{\overline{Y}}_{Rd}^{*} = \overline{y}_{ds} \left( {\frac{{\overline{x}_{ds}^{*} }}{{\overline{x}^{\prime } }}} \right),$$

Lone et al.^12^ worked out dual to ratio-cum-product type estimator in double sampling for stratification motivated by Singh^20^ and Lone et al.^17^ as

3.7 $$\hat{\overline{Y}}_{Rpd}^{*} = \;\overline{y}_{ds} \;\left( {\frac{{\overline{x}_{ds}^{*} }}{{\overline{x}^{\prime } }}} \right)\left( {\frac{{\overline{z}^{\prime } }}{{\overline{z}_{ds}^{*} }}} \right),$$

## Proposed estimator

Motivated by Ige and Tripathi^6^ and Tailor et al.^8^, we have developed an improved exponential type ratio estimator by assuming that $\overline{Z}$ is known and $\overline{x}^{\prime }$ is replaced by ratio-estimator $\overline{x}^{\prime } \exp \left( {\frac{{\overline{Z} - \overline{z}^{\prime } }}{{\overline{Z} + \overline{z}^{\prime } }}} \right)$, the developed estimator for estimating population mean $\overline{Y}$ is defined as

4.1 $$\hat{\overline{Y}}_{{C{\text{Re}} }}^{ds} = \left( {\frac{{\overline{y}_{ds} }}{{\overline{x}_{ds} }}} \right)\,\overline{x}^{\prime } \exp \left( {\frac{{\overline{Z} - \overline{z}^{\prime } }}{{\overline{Z} + \overline{z}^{\prime } }}} \right)$$

where $\overline{x}_{ds} \, = \,\sum\nolimits_{h = 1}^{L} {\,w_{h} \overline{x}_{h} }$: is unbiased estimator of $\overline{X}$ in second phase, $\overline{y}_{ds} \, = \,\,\sum\nolimits_{h = 1}^{L} {\,w_{h} } \overline{y}_{h}$: is unbiased estimator of $\overline{Y}$ in second phase,

The expression of bias and *MSE* of $\hat{\overline{Y}}_{{C{\text{Re}} }}^{ds}$ can be easily find by considering error terms *e*_*i*_ in such a way that

$$\overline{y}_{ds} = \overline{Y}\left( {1 + e_{o} } \right),$$

$$\overline{x}_{ds} = \overline{X}\left( {1 + e_{1} } \right),$$

$$\overline{x}^{\prime } = \overline{X}\left( {1 + e_{1}^{\prime } } \right)\,{\text{and}}$$

$$\overline{z}^{\prime } = \overline{Z}\left( {1 + e_{2}^{\prime } } \right)$$

Such that $E\,\left( {e_{o} } \right)\,\, = E(e_{1} ) = \,\,E\left( {e_{1}^{\prime } } \right)\,\, = E\,\,\left( {e_{2}^{\prime } } \right)\, = 0$ and

$$E\left( {e_{0}^{2} } \right) = \frac{1}{{\overline{Y}^{2} }}\left[ {S_{y\,}^{2} \left( {\frac{1 - f}{{n^{\prime } }}} \right) + \frac{1}{{n^{\prime } }}\sum\limits_{h = 1}^{L} {W_{h} \,S_{yh}^{2} \left( {\frac{1}{{v_{h} }} - 1} \right)} } \right]\,,$$

$$E\left( {e_{1}^{\prime 2} } \right) = \frac{1}{{\overline{X}^{2} }}S_{x\,}^{2} \left( {\frac{1 - f}{{n^{\prime}}}} \right),$$

$$E\left( {e_{1}^{2} } \right) = \frac{1}{{\overline{X}^{2} }}\left[ {S_{x\,}^{2} \left( {\frac{1 - f}{{n^{\prime } }}} \right) + \frac{1}{{n^{\prime } }}\sum\limits_{h = 1}^{L} {W_{h} \,S_{xh}^{2} \left( {\frac{1}{{v_{h} }} - 1} \right)} } \right]\,,$$

$$E\left( {e_{2}^{\prime 2} } \right) = \frac{1}{{\overline{Z}^{2} }}S_{z\,}^{2} \left( {\frac{1 - f}{{n^{\prime}}}} \right),$$

$$E\left( {e_{0}^{{}} e_{1} } \right) = \frac{1}{{\overline{Y}\,\overline{X}}}\left[ {\left( {\frac{1 - f}{{n^{\prime}}}} \right)S_{yx}^{{}} + \frac{1}{{n^{\prime}}}\sum\limits_{h = 1}^{L} {W_{h} \,S_{yxh}^{{}} \left( {\frac{1}{{v_{h} }} - 1} \right)} } \right]\,,$$

$$E\left( {e_{1}^{{}} e_{1}^{\prime } } \right) = \frac{1}{{\,\overline{X}^{2} }}S_{x\,}^{2} \left( {\frac{1 - f}{{n^{\prime } }}} \right),$$

$$E\left( {e_{0}^{{}} e_{1}^{\prime } } \right) = \frac{1}{{\overline{Y}\,\overline{X}}}\left( {\frac{1 - f}{{n^{\prime } }}} \right)S_{yx\,}^{{}} ,$$

$$E\left( {e_{0}^{{}} e_{2}^{\prime } } \right) = \frac{1}{{\overline{Y}\,\overline{Z}}}\left( {\frac{1 - f}{{n^{\prime}}}} \right)S_{yz\,}^{{}} ,$$

$$E\left( {e_{1} e_{2}^{\prime } } \right) = \frac{1}{{\overline{X}\,\overline{Z}}}\left( {\frac{1 - f}{{n^{\prime}}}} \right)S_{xz\,}^{{}} ,$$

$$E\left( {e_{1}^{\prime } e_{2}^{\prime } } \right) = \frac{1}{{\,\overline{X}\,\overline{Z}}}\left( {\frac{1 - f}{{n^{\prime}}}} \right)S_{xz\,}^{{}} .$$

where *e*_*i*_’s are error terms.

Substituting these values in (4.1), the developed estimator $\hat{\overline{Y}}_{{C{\text{Re}} }}^{ds}$ becomes

$$\hat{\overline{Y}}_{{C{\text{Re}} }}^{ds} = \overline{Y}\left( {1 + e_{0} } \right)\left( {1 + e_{1} } \right)^{ - 1} \left( {1 + e_{1}^{\prime } } \right)\exp \left\{ {\left( {\frac{{ - e_{2}^{\prime } }}{2}} \right)\left( {1 + \frac{{e_{2}^{\prime } }}{2}} \right)^{ - 1} } \right\}$$

4.2 $$\hat{\overline{Y}}_{{C{\text{Re}} }}^{ds} - \overline{Y} = \overline{Y}\left( {e_{0} - e_{1} + e_{1}^{\prime } - \frac{{e_{2}^{\prime } }}{2} - e_{0} e_{1} - e_{1} e_{1}^{\prime } + e_{0} e_{1}^{\prime } + e_{1}^{2} - \frac{{e_{0} e_{2}^{\prime } }}{2} + \frac{{e_{1} e_{2}^{\prime } }}{2} - \frac{{e_{1}^{\prime } e_{2}^{\prime } }}{2} + \frac{{3e_{2}^{\prime } }}{8}^{2} } \right)$$

Expectations of (4.2) proceeds towards the bias of $\hat{\overline{Y}}_{{C{\text{Re}} }}^{ds}$ and finally, up to the first degree of approximation (*fda*), the bias is obtained as

4.3 $$B\left( {\hat{\overline{Y}}_{{C{\text{Re}} }}^{ds} } \right) = \left[ {\frac{1}{{n^{\prime}\overline{X}}}\sum\limits_{i = 1}^{L} {W_{h} \left( {\frac{1}{{\nu_{h} }} - 1} \right)\left( {R_{1} S_{xh}^{2} - S_{yxh} } \right) + \frac{1}{{8\overline{Z}}}\frac{{\left( {1 - f} \right)}}{n^{\prime}}} \left( {3R_{2} S_{z}^{2} - S_{yz} } \right)} \right]$$

To find *MSE* of the developed estimator, we square and take expectation of (4.2)

$$MSE\left( {\hat{\overline{Y}}_{{C{\text{Re}} }}^{ds} } \right) = \overline{Y}^{2} E\left[ {e_{0} - e_{1} + e_{1}^{\prime } - \frac{{e_{2}^{\prime } }}{2}} \right]^{2}$$

$$= \overline{Y}^{2} E\left( {e_{0}^{2} + e_{1}^{2} + e_{1}^{\prime 2} + \frac{{e_{2}^{\prime 2} }}{4} - 2e_{0} e_{1} - 2e_{1} e_{1}^{\prime } + 2e_{0} e_{1}^{\prime } - e_{0} e_{2}^{\prime } + e_{1} e_{2}^{\prime } - e_{1}^{\prime } e_{2}^{\prime } } \right)$$

Using expected values of *e*’s, the *MSE* of the developed estimator $\hat{\overline{Y}}_{{C{\text{Re}} }}^{ds}$ is obtained up to *fda* as

$$MSE\left( {\overset{\lower0.5em\hbox{$\smash{\scriptscriptstyle\frown}$}}{\overline{Y}}_{{C{\text{Re}} }}^{ds} } \right) = \left( {\frac{1 - f}{{n^{\prime}}}} \right)S_{y}^{2} + \frac{1}{n^{\prime}}\sum\limits_{h = 1}^{L} {W_{h} \left( {\frac{1}{{\nu_{h} }} - 1} \right)} \left( {S_{yh}^{2} + R_{1}^{2} S_{xh}^{2} - 2R_{1}^{{}} S_{yxh}^{{}} } \right)$$

4.4 $$+ \frac{1}{4}\left( {\frac{1 - f}{{n^{\prime}}}} \right)\left( {R_{2}^{2} S_{z}^{2} - 4R_{2} S_{yz} } \right)$$

## Comparison with relevant estimators

From an efficiency perspective, the proposed estimator is compared to all other estimators discussed in Section “Some relevant existing estimators”. The variance of an unbiased estimator, the MSEs of an Ige and Tripathi^6^, Tailor et al.^8^, Lakhre^16^, Lone et al.^17^ and Lone et al.^12^ estimator are all provided in *DSS* as

5.1 $$V\left( {\overline{y}_{ds} } \right)= S_{y\,}^{2} \left( {\frac{1 - f}{{n^{\prime}}}} \right) + \frac{1}{{n^{\prime}}}\sum\limits_{h = 1}^{L} {W_{h} \,S_{yh}^{2} \left( {\frac{1}{{v_{h} }} - 1} \right)} ,$$

5.2 $$MSE\left( {\hat{\overline{Y}}_{R}^{ds} } \right) = S_{y}^{2} \left( {\frac{1 - f}{{n^{\prime } }}} \right) + \frac{1}{{n^{\prime } }}\sum\limits_{h = 1}^{L} {W_{h} \left( {\frac{1}{{\nu_{h} }} - 1} \right)} \left( {S_{yh}^{2} + R_{1}^{2} S_{xh}^{2} - 2R_{1} S_{yxh} } \right),$$

5.3 $$MSE\left( {\hat{\overline{Y}}_{{\text{Re}}}^{ds} } \right) = S_{y}^{2} \left( {\frac{1 - f}{{n{\prime} }}} \right) + \frac{1}{{n{\prime} }}\sum\limits_{h = 1}^{L} {W_{h} \left( {\frac{1}{{v_{h} }} - 1} \right)\left[ {S_{yh}^{2} + \frac{{R_{1}^{2} }}{4}S_{xh}^{2} \left( {1 - \frac{{\beta_{yxh} }}{{R_{1} }}} \right)} \right]} ,$$

5.4 $$MSE(\hat{\overline{Y}}_{{\text{Re}}}^{*} ) = S_{y}^{2} \left( {\frac{1 - f}{{n^{\prime } }}} \right)\; + \frac{1}{{n^{\prime } }}\sum\limits_{h = 1}^{L} {W_{h} \left( {\frac{1}{{\nu_{h} }} - 1} \right)} \;\left[ {S_{yh}^{2} + \;\frac{1}{4}R_{1}^{2} g^{2} S_{xh}^{2} \; - gR_{1} S_{yxh} } \right],$$

5.5 $$MSE(\hat{\overline{Y}}_{Rd}^{*} )\; = \;S_{y}^{2} \left( {\frac{1 - f}{{n^{\prime } }}} \right)\; + \;\frac{1}{{n^{\prime } }}\sum\limits_{h = 1}^{L} {W_{h} } \left( {\frac{1}{{\nu_{h} }} - 1} \right)\;\left[ {S_{yh}^{2} \; + g^{2} R_{1}^{2} S_{xh}^{2} \; - 2gR_{1} S_{yxh} } \right],$$

5.6 $$MSE(\hat{\overline{Y}}_{Rpd}^{*} )\; = \;S_{y}^{2} \left( {\frac{1 - f}{{n^{\prime } }}} \right)\; + \frac{1}{{n^{\prime } }}\sum\limits_{h = 1}^{L} {W_{h} } \left( {\frac{1}{{\nu_{h} }} - 1} \right)\;\left[ \begin{gathered} S_{yh}^{2} \; + g^{2} R_{1}^{2} S_{xh}^{2} \; + g^{2} R_{2}^{2} S_{zh}^{2} \hfill \\ - 2gR_{1} S_{yxh} + 2gR_{2} S_{yzh} - 2g^{2} R_{1} R_{2} S_{xzh} \hfill \\ \end{gathered} \right],$$

where $$g = \frac{n}{N - n}$$.

when (4.4), (5.1), (5.2), (5.3), (5.4), (5.5) and (5.6) are compared, it is clear that the developed chain ratio type exponential estimator would be more efficient than

(i) $$\overline{y}_{ds} \,\,\,if$$
  5.7 $$\left( {1 - f} \right)\;\left( {R_{2}^{2} S_{z}^{2} - 4R_{2} S_{yz} } \right)\; \le \;\sum\limits_{h = 1}^{L} {W_{h} \left( {\frac{1}{{\nu_{h} }} - 1} \right)\;\left( {8R_{1} S_{xh}^{2} - 4R_{1}^{2} S_{xh}^{2} } \right)} \;,$$
(ii) Ige and Tripathi^6^ estimator $\hat{\overline{Y}}_{R}^{ds}$ if
  5.8 $$S_{z}^{2} \; \le \;\frac{{4S_{yz} }}{{R_{2} }}\;\;,$$
(iii) Tailor et al.^8^ estimator $\hat{\overline{Y}}_{Re}^{ds}$ if
  5.9 $$\left( {1 - f} \right)\;\left( {R_{2}^{2} S_{z}^{2} - 4R_{2} S_{yz} } \right)\; \le \;\;\sum\limits_{h = 1}^{L} {W_{h} \left( {\frac{1}{{\nu_{h} }} - 1} \right)\;\left( {8R_{1} S_{yxh} - 8R_{1} S_{xh}^{2} - R_{1} S_{xh}^{2} \beta_{yxh} } \right)} \;,$$
(iv) Lakhre^16^ estimator $\hat{\overline{Y}}_{{\text{Re}}}^{*}$ if
  5.10 $$\left( {1 - f} \right)\;\left( {R_{2}^{2} S_{z}^{2} - 4R_{2} S_{yz} } \right)\; \le \;4\;\left[ {\sum\limits_{h = 1}^{L} {W_{h} \left( {\frac{1}{{\nu_{h} }} - 1} \right)\;\left( {R_{1}^{2} S_{xh}^{2} \left( {\frac{{g^{2} }}{4} - 1} \right) + R_{1} S_{yxh} \left( {2 - g} \right)} \right)} } \right],$$
(v) Lone et al.^17^ estimator $\hat{\overline{Y}}_{Rd}^{*}$ if
  5.11 $$\left( {1 - f} \right)\;\left( {R_{2}^{2} S_{z}^{2} - 4R_{2} S_{yz} } \right)\; \le \;4\;\left[ {\sum\limits_{h = 1}^{L} {W_{h} \left( {\frac{1}{{\nu_{h} }} - 1} \right)\;\left( {R_{1}^{2} S_{xh}^{2} \left( {g^{2} - 1} \right) + 2R_{1} S_{yxh} \left( {1 - g} \right)} \right)} } \right],$$
(vi) Lone et al.^12^ estimator $\hat{\overline{Y}}_{Rpd}^{*}$ if
  5.12 $$\left( {1 - f} \right)\;\left( {R_{2}^{2} S_{z}^{2} - 4R_{2} S_{yz} } \right)\; \le \;4\;\left[ {\sum\limits_{h = 1}^{L} {W_{h} \left( {\frac{1}{{\nu_{h} }} - 1} \right)\;\left( \begin{gathered} R_{1} S_{xh}^{2} \left( {g^{2} - 1} \right) + 2R_{1} S_{yxh} \left( {1 - g} \right) + g^{2} R_{2}^{2} S_{zh}^{2} \hfill \\ + 2gR_{2} S_{yzh} - 2g^{2} R_{1} R_{2} S_{xzh} \hfill \\ \end{gathered} \right)} } \right].$$

## Empirical study

In Section “Comparison with relevant estimators”, the developed chain ratio type exponential estimator was compared theoretically. In this section numerical illustration is being discussed to show the performance of different considered estimators as well as the proposed estimator practically and the percent relative efficiency (PRE) of the proposed estimator compared to other considered estimators is also shown in Table 2. For this purpose, two data sets have been considered. Description of data set is given below:

**Table:** 

Variables	Population I- [Source: Singh and Chaudhary^22^, P. 177]		Population II- [Source: Murthy^21^, p. 228]	
$y$	Productivity		Output	
$x$	Production		Fixed capital	
$z$	Area		Number of workers	

	Population I		Population II	
Parameters	Stratum I	Stratum II	Stratum I	Stratum II
$N_{h}$	10	10	5	5
$n_{h}$	4	4	2	2
$n^{\prime}_{h}$	6	6	4	4
$\overline{Y}_{h}$	264.00	214.70	1925.80	3115.60
$\overline{X}_{h}$	939.00	1121.50	214.40	333.80
$\overline{Z}_{h}$	263.20	202.90	51.80	60.60
$S_{yh}$	149.53	192.02	615.92	340.38
$S_{xh}$	389.67	1165.20	74.87	66.35
$S_{zh}$	162.85	178.54	0.75	4.84
$S_{yxh}$	53,277.00	68,650.00	39,360.68	22,356.50
$S_{yzh}$	23,798.00	33,841.00	411.16	1536.24
$S_{xzh}$	58,729.00	60,376.00	38.08	287.92
$S_{y}^{2}$	31,814.87		668,351.00	
$S_{z}^{2}$	31,692.05		34.84.00	
$S_{yz}$	29,562.58		1668.23	

## Simulation study

In this section, simulation study has been carried out to observe the performance of the developed estimator as compared to other considered estimators by using R-software. Six different pseudo populations of size $N$ having two strata $N_{1}$ and $N_{2}$ of equal and unequal sizes have been generated. All the populations are simulated from normal distribution. The values of PRE and MSE of the populations having equal strata sizes are given in Tables 3, 4 respectively and Tables 5 and 6 show PRE and MSE values of the populations having unequal strata sizes. The results of the simulated data sets are also represented with the help of line graphs, where it is clearly shown that the developed estimator has highest PRE and least MSE in each population.

**Populations having equal strata size:**

Population 1: $$N$$ = 800, $$N_{1}$$ = 400, $$N_{2}$$ = 400, $$n^{\prime }$$ = 600, $$n_{1}^{\prime }$$ = 300, $$n_{2}^{\prime }$$ = 300, $$n$$ = 300.

Population 2: $$N$$ = 500, $$N_{1}$$ = 250, $$N_{2}$$ = 250, $$n^{\prime }$$ = 350, $$n_{1}^{\prime }$$ = 175, $$n_{2}^{\prime}$$ = 175, $$n$$ = 174.

Population 3: $$N$$ = 1000, $$N_{1}$$ = 500, $$N_{2}$$ = 500, $$n^{\prime}$$ = 600, $$n_{1}^{\prime}$$ = 300, $$n_{2}^{\prime}$$ = 300, $$n$$ = 300.

**Populations having unequal strata size:**

Population 1: $$N$$ = 1500, $$N_{1}$$ = 650, $$N_{2}$$ = 850, $$n^{\prime}$$ = 1000, $$n_{1}^{\prime}$$ = 400, $$n_{2}^{\prime}$$ = 600, $$n$$ = 500.

Population 2: $$N$$ = 400, $$N_{1}$$ = 150, $$N_{2}$$ = 250, $$n^{\prime}$$ = 300, $$n_{1}^{\prime}$$ = 100, $$n_{2}^{\prime}$$ = 200, $$n$$ = 150.

Population 3: $$N$$ = 2000, $$N_{1}$$ = 1200, $$N_{2}$$ = 800, $$n^{\prime}$$ = 1200, $$n_{1}^{\prime}$$ = 800, $$n_{2}^{\prime}$$ = 400, $$n$$ = 500.

## Results and discussions

**Figure 1:**
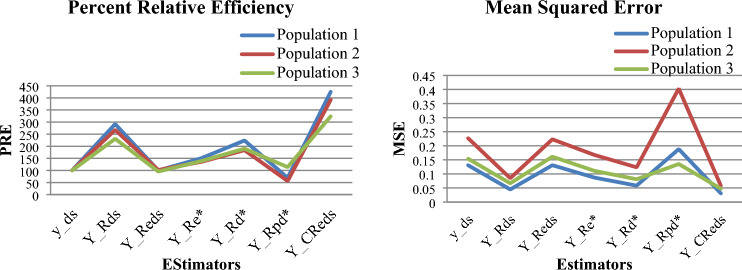
Graphs for percent relative efficiency and mean square error $\overline{y}_{ds}$, $\hat{\overline{Y}}_{R}^{ds}$, $\hat{\overline{Y}}_{{\text{Re}}}^{ds}$, $\hat{\overline{Y}}_{{\text{Re}}}^{*}$, $\hat{\overline{Y}}_{Rd}^{*}$, $\hat{\overline{Y}}_{Rpd}^{*}$ and $\hat{\overline{Y}}_{CRe}^{ds}$ with respect to $\overline{y}_{ds}$ for equal strata size.

**Figure 2:**
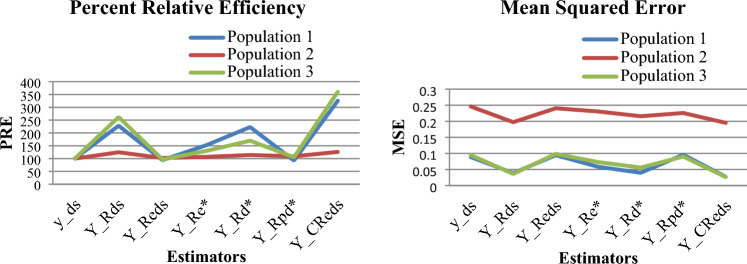
Graphs for percent relative efficiency and mean square error $\overline{y}_{ds}$, $\hat{\overline{Y}}_{R}^{ds}$, $\hat{\overline{Y}}_{{\text{Re}}}^{ds}$, $\hat{\overline{Y}}_{Rd}^{*}$, $\hat{\overline{Y}}_{{\text{Re}}}^{*}$, $\hat{\overline{Y}}_{Rpd}^{*}$ and $\hat{\overline{Y}}_{CRe}^{ds}$ with respect to $\overline{y}_{ds}$ for unequal strata size.

(i) Table 1 demonstrates that among the two real data sets, population 1 meets all the conditions outlined in Section “Comparison with relevant estimators”, under which the proposed estimator outperform other considered estimators. In contrast, population 2 fails to fulfill the conditions specified in Eqs. (5.11) and (5.12). Table 2 presents the percent relative efficiency (PRE) of all the considered estimators discussed in Section “Empirical study” as well as the proposed estimator for the two real data sets. The symbol ‘*’ is used to indicate instances where PRE is not applicable, as these conditions, as detailed in Eqs. (5.11) and (5.12), remain unsatisfied, a fact also highlighted in Table 1 (rows 5 and 6).
(ii) The PRE and MSE values of the proposed and other considered estimators for the first three simulated normal populations having equal strata sizes are given in Tables 3 and 4 respectively. Similarly, Tables 5 and 6 show the PRE and MSE values for the next three simulated normal populations having unequal strata sizes.
(iii) Figures 1 and 2 show the PRE and MSE of the first three simulated normal populations having equal strata sizes and the next three simulated normal populations having unequal strata sizes respectively.
(iv) It is observed from all the tables as well as from all the graphs that the proposed estimator has least MSE and highest PRE among other considered estimators which indicates that the proposed estimator will perform better for practical purpose compared to other considered estimators such as $\overline{y}_{ds}$, $\hat{\overline{Y}}_{R}^{ds}$, $\hat{\overline{Y}}_{{\text{Re}}}^{ds}$, $\hat{\overline{Y}}_{{\text{Re}}}^{*}$ $\hat{\overline{Y}}_{Rd}^{*}$ and $\hat{\overline{Y}}_{Rpd}^{*}$ under the conditions given in Section “Comparison with relevant estimators”.

**Table 1:** Empirical exhibition of theoretical conditions given in Sect. “Some relevant existing estimators”

Sr. No.	Comparison	Conditions	Population1	Population 2
_1_	$$\begin{gathered} MSE\left( {\overset{\lower0.5em\hbox{$\smash{\scriptscriptstyle\frown}$}}{\overline{Y}}_{{C{\text{Re}} }}^{ds} } \right) \hfill \\ < MSE\left( {\overline{y}_{ds} } \right) \hfill \\ \end{gathered}$$	$$\begin{gathered} \left( {1 - f} \right)\left( {R_{2}^{2} S_{z}^{2} - 4R_{2} S_{yz} } \right) < \hfill \\ \sum\limits_{{}}^{{}} {W_{h} \left( {\frac{1}{{\nu_{h} }} - 1} \right)\left( {8R_{1} S_{yxh} - 4R_{1}^{2} S_{xh}^{2} } \right)} \hfill \\ \end{gathered}$$	− 35,207.30 < − 24,822.30	− 45,841.40 < 577,546.89
_2_	$$\begin{gathered} MSE\left( {\overset{\lower0.5em\hbox{$\smash{\scriptscriptstyle\frown}$}}{\overline{Y}}_{{C{\text{Re}} }}^{ds} } \right) \hfill \\ < MSE\left( {\overline{y}_{R}^{ds} } \right) \hfill \\ \end{gathered}$$	$$S_{z}^{2} < \frac{{4S_{yz} }}{{R_{2} }}$$	31,692.05 < 1,15,137.80	34.84 < 148.77
_3_	$$\begin{gathered} MSE\left( {\overset{\lower0.5em\hbox{$\smash{\scriptscriptstyle\frown}$}}{\overline{Y}}_{{C{\text{Re}} }}^{ds} } \right) \hfill \\ < MSE\left( {\hat{\overline{Y}}_{{\text{Re}}}^{ds} } \right) \hfill \\ \end{gathered}$$	$$\begin{gathered} \left( {1 - f} \right)\left( {R_{2}^{2} S_{z}^{2} - 4R_{2} S_{yz} } \right) < \hfill \\ \sum\limits_{{}}^{{}} {W_{h} \left( {\frac{1}{{\nu_{h} }} - 1} \right)\left( {8R_{1} S_{yxh} - 3R_{1}^{2} S_{xh}^{2} - R_{1} S_{xh}^{2} \beta_{yxh} } \right)} \hfill \\ \end{gathered}$$	− 35,207.3 0 < − 11,535.13	− 45,841.40 < 716,948.13
_4_	$$\begin{gathered} MSE(\hat{\overline{Y}}_{{C{\text{Re}} }}^{ds} ) \hfill \\ < \;MSE(\hat{\overline{Y}}_{{\text{Re}}}^{*} ) \hfill \\ \end{gathered}$$	$$\begin{gathered} \left( {1 - f} \right)\;\left( {R_{2}^{2} S_{z}^{2} - 4R_{2} S_{yz} } \right)\; \le \; \hfill \\ 4\;\left[ {\sum\limits_{h = 1}^{L} {W_{h} \left( {\frac{1}{{\nu_{h} }} - 1} \right)\;\left( {R_{1}^{2} S_{xh}^{2} \left( {\frac{{g^{2} }}{4} - 1} \right) + R_{1} S_{yxh} \left( {2 - g} \right)} \right)} } \right] \hfill \\ \end{gathered}$$	− 35,207.32 < − 34,653.76	− 45,841.44 < 8842.96
_5_	$$\begin{gathered} MSE(\hat{\overline{Y}}_{{C{\text{Re}} }}^{ds} ) \hfill \\ < \;MSE(\hat{\overline{Y}}_{Rd}^{*} ) \hfill \\ \end{gathered}$$	$$\begin{gathered} \left( {1 - f} \right)\;\left( {R_{2}^{2} S_{z}^{2} - 4R_{2} S_{yz} } \right)\; \le \; \hfill \\ 4\;\left[ {\sum\limits_{h = 1}^{L} {W_{h} \left( {\frac{1}{{\nu_{h} }} - 1} \right)\;\left( {R_{1}^{2} S_{xh}^{2} \left( {g^{2} - 1} \right) + 2R_{1} S_{yxh} \left( {1 - g} \right)} \right)} } \right] \hfill \\ \end{gathered}$$	− 35,207.32 < − 26,379.66	*
_6_	$$\begin{gathered} MSE(\hat{\overline{Y}}_{{C{\text{Re}} }}^{ds} ) \hfill \\ < \;MSE(\hat{\overline{Y}}_{Rpd}^{*} ) \hfill \\ \end{gathered}$$	$$\begin{gathered} \left( {1 - f} \right)\;\left( {R_{2}^{2} S_{z}^{2} - 4R_{2} S_{yz} } \right)\; \le \hfill \\ \;4\;\left[ {\sum\limits_{h = 1}^{L} {W_{h} \left( {\frac{1}{{\nu_{h} }} - 1} \right)\;\left( \begin{gathered} R_{1} S_{xh}^{2} \left( {g^{2} - 1} \right) \hfill \\ + 2R_{1} S_{yxh} \left( {1 - g} \right) + g^{2} R_{2}^{2} S_{zh}^{2} \hfill \\ + 2gR_{2} S_{yzh} - 2g^{2} R_{1} R_{2} S_{xzh} \hfill \\ \end{gathered} \right)} } \right] \hfill \\ \end{gathered}$$	− 35,207.32 < 54,665.01	*

“*” conditions not satisfied.

**Table 2:** Percent relative efficiencies of $$\overline{y}_{ds}$$, $$\hat{\overline{Y}}_{R}^{ds}$$, $$\hat{\overline{Y}}_{{\text{Re}}}^{ds}$$, $$\hat{\overline{Y}}_{{\text{Re}}}^{*}$$$$\hat{\overline{Y}}_{Rd}^{*}$$, $$\hat{\overline{Y}}_{Rpd}^{*}$$ and $$\hat{\overline{Y}}_{CRe}^{ds}$$ with respect to $$\overline{y}_{ds}$$.

Estimators	Percent relative efficiency	
	Population 1	Population 2
$\overline{y}_{ds}$	100.00	100.00
$\hat{\overline{Y}}_{R}^{ds}$	81.60	160.95
$\hat{\overline{Y}}_{{\text{Re}}}^{ds}$	89.23	91.62
$\hat{\overline{Y}}_{{\text{Re}}}^{*}$	109.80	159.45
$\hat{\overline{Y}}_{Rd}^{*}$	101.43	*
$\hat{\overline{Y}}_{Rpd}^{*}$	58.08	*
$\hat{\overline{Y}}_{{C{\text{Re}} }}^{ds}$	**110.41**	**169.13**

“*” Not applicable.

Significant values are in bold.

**Table 3:** Percent relative efficiencies of $\overline{y}_{ds}$, $\hat{\overline{Y}}_{R}^{ds}$, $\hat{\overline{Y}}_{{\text{Re}}}^{ds}$, $\hat{\overline{Y}}_{{\text{Re}}}^{*}$, $\hat{\overline{Y}}_{Rd}^{*}$, $\hat{\overline{Y}}_{Rpd}^{*}$ and $\hat{\overline{Y}}_{CRe}^{ds}$ with respect to $\overline{y}_{ds}$ for equal strata size.

Estimators	Percent relative efficiency		
	Population 1	Population 2	Population 3
$\overline{y}_{ds}$	100.00	100.00	100.00
$\hat{\overline{Y}}_{R}^{ds}$	291.93	267.45	230.95
$\hat{\overline{Y}}_{{\text{Re}}}^{ds}$	99.94	101.74	95.51
$\hat{\overline{Y}}_{{\text{Re}}}^{*}$	150.66	135.63	138.91
$\hat{\overline{Y}}_{Rd}^{*}$	223.89	183.75	190.02
$\hat{\overline{Y}}_{Rpd}^{*}$	69.52	56.24	113.86
$\hat{\overline{Y}}_{{C{\text{Re}} }}^{ds}$	**425.27**	**392.40**	**323.96**

Significant values are in bold.

**Table 4:** Mean squared error of $\overline{y}_{ds}$, $\hat{\overline{Y}}_{R}^{ds}$, $\hat{\overline{Y}}_{{\text{Re}}}^{ds}$, $\hat{\overline{Y}}_{{\text{Re}}}^{*}$, $\hat{\overline{Y}}_{Rd}^{*}$, $\hat{\overline{Y}}_{Rpd}^{*}$ and $\hat{\overline{Y}}_{CRe}^{ds}$ with respect to $\overline{y}_{ds}$ for equal strata size.

Estimators	Mean squared error		
	Population 1	Population 2	Population 3
$\overline{y}_{ds}$	0.1308	0.2268	0.1539
$\hat{\overline{Y}}_{R}^{ds}$	0.0448	0.0848	0.0666
$\hat{\overline{Y}}_{{\text{Re}}}^{ds}$	0.1308	0.2229	0.1611
$\hat{\overline{Y}}_{{\text{Re}}}^{*}$	0.0868	0.1672	0.1108
$\hat{\overline{Y}}_{Rd}^{*}$	0.0584	0.1234	0.0810
$\hat{\overline{Y}}_{Rpd}^{*}$	0.1881	0.4032	0.1352
$\hat{\overline{Y}}_{{C{\text{Re}} }}^{ds}$	**0.0307**	**0.0578**	**0.0475**

Significant values are in bold.

**Table 5:** Percent relative efficiencies of $\overline{y}_{ds}$, $\hat{\overline{Y}}_{R}^{ds}$, $\hat{\overline{Y}}_{{\text{Re}}}^{ds}$, $\hat{\overline{Y}}_{{\text{Re}}}^{*}$, $\hat{\overline{Y}}_{Rd}^{*}$, $\hat{\overline{Y}}_{Rpd}^{*}$ and $\hat{\overline{Y}}_{CRe}^{ds}$ with respect to $\overline{y}_{ds}$ for unequal strata size.

Estimators	Percent relative efficiency		
	Population 1	Population 2	Population 3
$\overline{y}_{ds}$	100.00	100.00	100.00
$\hat{\overline{Y}}_{R}^{ds}$	228.17	124.69	262.03
$\hat{\overline{Y}}_{{\text{Re}}}^{ds}$	93.90	102.30	96.342
$\hat{\overline{Y}}_{{\text{Re}}}^{*}$	152.24	106.74	130.06
$\hat{\overline{Y}}_{Rd}^{*}$	222.79	114.03	169.78
$\hat{\overline{Y}}_{Rpd}^{*}$	92.49	108.94	105.23
$\hat{\overline{Y}}_{{C{\text{Re}} }}^{ds}$	**325.85**	**126.06**	**360.46**

Significant values are in bold.

**Table 6:** Mean squared error of $\overline{y}_{ds}$, $\hat{\overline{Y}}_{R}^{ds}$, $\hat{\overline{Y}}_{{\text{Re}}}^{ds}$, $\hat{\overline{Y}}_{{\text{Re}}}^{*}$, $\hat{\overline{Y}}_{Rd}^{*}$, $\hat{\overline{Y}}_{Rpd}^{*}$ and $\hat{\overline{Y}}_{CRe}^{ds}$ with respect to $\overline{y}_{ds}$ for unequal strata size.

Estimators	Mean squared error		
	Population 1	Population 2	Population 3
$\overline{y}_{ds}$	0.0886	0.2460	0.0949
$\hat{\overline{Y}}_{R}^{ds}$	0.0388	0.1973	0.0362
$\hat{\overline{Y}}_{{\text{Re}}}^{ds}$	0.0944	0.2405	0.0985
$\hat{\overline{Y}}_{{\text{Re}}}^{*}$	0.0582	0.2305	0.0729
$\hat{\overline{Y}}_{Rd}^{*}$	0.0397	0.2157	0.0559
$\hat{\overline{Y}}_{Rpd}^{*}$	0.0958	0.2258	0.0902
$\hat{\overline{Y}}_{{C{\text{Re}} }}^{ds}$	**0.0272**	**0.1951**	**0.0263**

Significant values are in bold.

## Conclusion

In this study, we have investigated the problem of estimating the population mean of the study variable. We have proposed a chain ratio-type exponential estimator and examined its properties such as bias and mean squared error up to the first degree of approximation. Our analysis in Section “Comparison with relevant estimators” has established the conditions under which the proposed estimator outperforms all other estimators shown in Section ““Some relevant existing estimators””. The empirical as well as simulation study have been conducted to support the theoretical findings. The proposed estimator found to be more efficient compared to other considered estimators under some conditions given in Section ““Comparison with relevant estimators”” as it has least MSE and highest PRE among other estimators. The results are shown with the help of Tables 1, 2, 3, 4, 5 and 6 and also by using graphs shown in Figs. 1 and 2.

Overall, our research contributes significantly to the theory of estimating the population mean in the context of double sampling for stratification. Therefore, we recommend the application of our proposed estimator for the estimation of population mean in real-life situations.

## Data Availability

All the necessary data generated and/or analyzed during the current study are included in this published article.

## References

[1] Hosking JR (1990). L-moments: Analysis and estimation of distributions using linear combinations of order statistics. J. R. Stat. Soc. Ser. B.

[2] Shahzad U, Ahmad I, Almanjahie IM, Al–Noor NH (2021). L-moments based calibrated variance estimators using double stratified sampling. Comput. Mater. Contin..

[3] Lam TY, Kleinn C, Coenradie B (2011). Double sampling for stratification for the monitoring of sparse tree populations: the example of Populus euphratica Oliv. Forests at the lower reaches of Tarim River, Southern Xinjiang, China. Environ. Monit. Assess..

[4] Neyman J (1938). Contribution to the theory of sampling human population. J. Am. Stat. Assoc..

[5] Rao JNK (1973). On double sampling for stratification and analytical surveys. Biometrika.

[6] Ige AF, Tripathi TP (1987). On doubling for stratification and use of auxiliary information. J. Indian Soc. Agricult. Stat..

[7] Singh HP, Vishwakarma GK (2007). A general procedure for estimating the mean using double sampling for stratification. Model Assist. Stat. Appl..

[8] Tailor R, Chouhan S, Kim JM (2014). Ratio and product type exponential estimators of population mean in double sampling for stratification. Commun. Stat. Appl. Methods.

[9] Tailor R, Lone HA (2014). Ratio-cum-product estimator of finite population mean in double sampling for stratification. J. Reliab. Stat. Stud..

[10] Singh HP, Nigam P (2020). Ratio-ratio-type exponential estimator of finite population mean in double sampling for stratification. Int. J. Agricult. Stat. Sci..

[11] Gupta A, Tailor R (2021). Ratio in ratio type exponential strategy for the estimation of population mean. J. Reliab. Stat. Stud..

[12] Lone HA, Tailor R, Verma MR (2022). A note on the estimation of population mean in double sampling for stratification. J. Indian Soc. Agricult. Stat..

[13] Verma MR, Lone HA, Tailor R (2023). Generalized dual to ratio-cum-product type estimators in double sampling for stratification. J. Indian Soc. Agricult. Stat..

[14] Cochran WG (1940). The estimation of the yields of the cereal experiments by sampling for the ratio of grain to total produce. J. Agricult. Sci..

[15] Bahl S, Tuteja RK (1991). Ratio and product type exponential estimators. J. Inf. Optim. Sci..

[16] Lakhre A (2017). Dual to ratio and product type exponential estimators of finite population mean in double sampling for stratification. Int. J. Sci. Res. Math. Stat. Sci..

[17] Lone HA, Tailor R, Verma MR (2020). An alternative to ratio and product type estimators of finite population mean in double sampling for stratification. J. Indian Soc. Agricult. Stat..

[18] Srivenkataramana T (1980). A dual of ratio estimator in sample surveys. Biometrika.

[19] Bandyopadhyay S (1980). Improved ratio and product estimators. Sankhya SeriesC.

[20] Singh MP (1967). Ratio cum product method of estimation. Metrika.

[21] Murthy MN (1967). Sampling Theory and Methods.

[22] Singh D, Chaudhary FS (1971). Theory and Analysis of Sample Survey Designs.

